# Left and Right Lung Asynchrony as a Physiological Indicator for Unilateral Bronchial Obstruction in Interventional Bronchoscopy

**DOI:** 10.1371/journal.pone.0105327

**Published:** 2014-08-18

**Authors:** Masamichi Mineshita, Hirotaka Kida, Hiroki Nishine, Hiroshi Handa, Takeo Inoue, Teruomi Miyazawa

**Affiliations:** Division of Respiratory and Infectious Diseases, Department of Internal Medicine, St. Marianna University School of Medicine, Kawasaki, Kanagawa, Japan; National Institute of Environmental Health Sciences, United States of America

## Abstract

**Background:**

In patients with bronchial obstruction, pulmonary function tests may not change significantly after intervention. The airflow asynchrony in both lungs due to unilateral bronchial obstruction may be applicable as a physiological indicator. The airflow asynchrony is reflected by the difference in the left and right lung sound development at tidal breathing.

**Objectives:**

To investigate the usefulness of left and right lung asynchrony due to unilateral bronchial obstruction as a physiological indicator for interventional bronchoscopy.

**Methods:**

Fifty cases with central airway obstruction were classified into three groups: tracheal, bronchial and extensive obstruction. The gap index was defined as the absolute value of the average of gaps between the left and right lung sound intensity peaks for a 12-second duration.

**Results:**

Before interventional bronchoscopy, the gap index was significantly higher in the bronchial (p<0.05) and extensive obstruction groups (p<0.05) than in the tracheal group. The gap index in cases with unilateral bronchial obstruction of at least 80% (0.18±0.04 seconds) was significantly higher than in cases with less than 80% obstruction (0.02±0.01 seconds, p<0.05). After intervention for bronchial obstruction, the dyspnea scale (p<0.001) and gap index significantly improved (p<0.05), although no significant improvements were found in spirometric assessments. The responder rates for dyspnea were 79.3% for gap indexes over 0.06 seconds and 55.6% for gap indexes of 0.06 seconds or under.

**Conclusions:**

Assessment of left and right lung asynchrony in central airway obstruction with bronchial involvement may provide useful physiological information for interventional bronchoscopy.

## Introduction

Patients suffering from central airway obstruction (CAO) can receive significant relief from life-threatening symptoms through interventional bronchoscopy. Performing interventional procedures at the exact location of the obstruction can provide the greatest functional benefit to patients [1]–[10].

We have applied multi-modal, objective assessments using technologies such as endobronchial ultrasonography, three-dimensional computed tomography scans, and impulse oscillation systems (IOS) to provide useful information on collapsible segments and choke points in CAO patients [10]–[14]. To evaluate the effects of intervention during the procedure, we have recently introduced airway pressure assessment. The lateral airway pressure is measured during intervention to physiologically evaluate tracheal obstruction, and we have found that lateral airway pressure measurement can estimate the need for additional procedures [15]. The abovementioned measurements provide valuable visual and physiological information on CAO that is essential for a favorable outcome after intervention.

Regarding physiological assessment, we have noticed distinct flow-volume curve patterns specific to the type of obstruction [10]. Although flow-volume curves are widely used and easy to perform in daily clinical settings, they cannot discriminate between the left and right lungs. Furthermore, in patients with bronchial obstruction, pulmonary function tests may not change significantly after intervention [16].

In patients with CAO, airflow in the lungs is severely disturbed by the obstructed airway. The airflow asynchrony in both lungs due to unilateral bronchial obstruction may be applicable as a physiological indicator in bronchial obstruction. Airflow asynchrony may be evaluated by the difference in the left and right lung respiratory sound development in tidal breathing.

Airflow in the lung vibrates the airway wall and produces lung sounds. A correlation between lung sound recordings and the regional distribution of pulmonary ventilation has been reported, particularly in studies comparing acoustic findings with data obtained with radioactive gases in normal subjects [16], [17]. Recently, Shi et al. reported that measurements of regional ventilation distribution by lung sound were comparable to those obtained by electrical impedance tomography in a piglet model [18].

Lung sound recordings are evaluated quantitatively and qualitatively by computer software [18]–[27]. We reported that the location of the CAO and procedural outcomes were reliably identified according to specific patterns of lung sound distribution. Especially in unilateral bronchial obstruction, there was a consistent difference in the lung sound dynamics and intensity between the lungs. We found that the development of lung sound intensity of the obstructed side lagged behind that of the preserved side [19].

In bronchial asthma, asynchrony of the left and right lung sounds were detected by analyzing the gap between intensity peaks [25]. We speculated that analysis of left and right lung asynchrony using lung sound analysis might be a useful marker for main bronchial obstruction and as an outcome measurement for interventional bronchoscopy.

## Methods

Between May 2007 and December 2011, we performed a prospective study that was approved by the Research Ethics Committee at St. Marianna University School of Medicine. Written informed consent was obtained from all patients.

Adult patients previously diagnosed with CAO were enrolled and classified into three groups: tracheal, bronchial and extensive obstruction (defined as an obstruction extending from the trachea, including the carina, to the bronchi). Lung sound analysis, pulmonary function tests (PFTs), and the modified Medical Research Council (MMRC) scale were measured before and after intervention.

For lung sound analysis, the vibration response imaging (VRI) device (VRIxp System, Deep Breeze, Or-Akiva, Israel) was used as previously described [19]–[22], [26], [27]. The VRIxp System is a computer-based acoustic lung imaging platform that was developed to acquire, quantify, monitor and store breath sounds. Breath sounds were recorded with 7-row arrays; 6-row arrays were used if the height of the subject was less than 165 cm. Each subject was seated in a quiet environment with their hands resting in their laps. The right and left planar arrays were placed symmetrically on the subject's back using a low-vacuum, computer-controlled method. The two bottom-row arrays were positioned at approximately the same height as the two arrays that were parallel to the vertebral column. The recording was performed over a 12-second period while the subject took deep, regular breaths at a rate of 15–20 breaths per minute. Each subject was recorded at least three times, and the VRI recording with the highest technical quality was chosen for evaluation. Lung sound intensity was plotted over time (12 seconds) for each lung, with time on the x-axis and decibels on the y-axis. The timing of the inspiratory and expiratory lung sound intensity peaks was compared for the left and right lungs [25]. The gap index was defined as the absolute value of the average of gaps (seconds) between the lung sound intensity peaks of both lungs for the duration of 12 seconds.

Multi-detector computed tomography (MDCT) was performed with a 64-detector row CT scanner (Aquilion-64, Toshiba Medical Systems, Otawara, Japan) as previously described [28], [29]. The degree of bronchial obstruction was defined using the following formula, in which “CSA” stands for “cross-sectional area”: (CSA of less stenotic main bronchus – CSA of more stenotic main bronchus) / CSA of less stenotic main bronchus [15].

### Statistical Analysis

Data are reported as mean ± standard error (SE) unless otherwise indicated. All analyses were performed using SAS software (Release 9.2; SAS Institute, Cary, NC, USA). Significance tests were two-sided, and p values of 0.05 or less were defined as significant. One-way ANOVA with Dunnett adjustment was used to evaluate the difference in pulmonary function tests and lung sound recordings between tracheal obstruction group and CAO with bronchial involvement (bronchial and extensive obstruction group). Significant differences in MMRC, PFTs and lung sound recordings before and after intervention were evaluated using the Wilcoxon signed-rank test. The Mann-Whitney test was used to evaluate the significant difference in the gap index between two different severity stages of bronchial obstruction.

## Results

### Patient characteristics

Fifty cases of CAO were included in this study. There were 34 male and 16 female participants with a mean age of 55.6 years. CAO cases were diagnosed by bronchoscopy and chest CT and classified into three groups: tracheal (n = 12), bronchial (n = 18) and extensive obstruction (n = 20). Thirty-six cases were treated with combination therapy, including balloon dilatation, ablation, and stenting; 11 were treated with balloon dilation alone; and 3 were treated with mechanical resection alone (Table 1).

**Table 1:** Characteristics of patients with central airway obstruction.

		Type of central airway obstruction n = 50		
		Tracheal	Bronchial	Extensive
		n = 12	n = 18	n = 20
Age, yr	Mean	57.5	51.7	58.1
	Range	35–77	17–71	34–75
Sex	Male	9	11	14
	Female	3	7	6
Diagnosis				
Lung cancer		4	5	11
Esophageal cancer		2		2
Thyroid cancer		1		
Metastasis of colon cancer		1		
Germinal tumor		1		
Tuberculosis			8	4
Wegener granulomatosis			3	
Relapsing polychondoritis				2
Post intubation tracheal obstruction		2		
Others		1	2	1

Lung sounds and pulmonary function tests from 50 cases were recorded before and after intervention.

### MMRC, PFTs and lung sound recordings before and after interventional bronchoscopy

**Figure 1:**
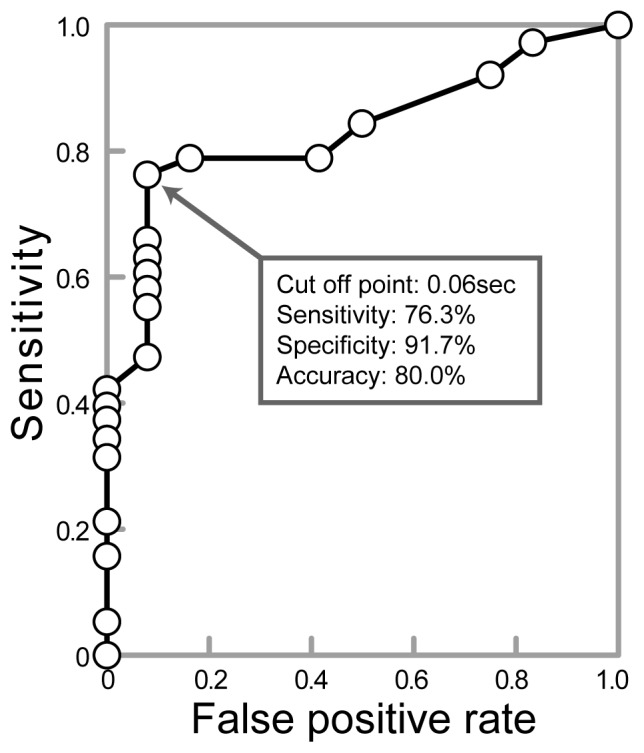
Receiver operating characteristic (ROC) validation of the gap index in the diagnosis of bronchial involvement in patients with central airway obstruction (CAO). ROC validation of the gap index revealed that a cut-off point of 0.06 seconds showed 76.3% sensitivity, 91.7% specificity, and 80% accuracy in the differentiation between CAO with bronchial involvement (bronchial = 18 and extensive = 20) and tracheal obstruction (n = 12).

For lung sound recordings before intervention, gap indexes for bronchial and extensive obstruction cases were significantly higher than those of tracheal obstruction (Table 2; tracheal: 0.039±0.011 seconds; bronchial: 0.216±0.050 seconds, p<0.05; extensive: 0.190±0.044 seconds, p<0.05). Receiver operating characteristics (ROC) validation of the gap index revealed that a cut-off point of 0.06 seconds showed 76.3% sensitivity, 91.7% specificity, and 80% accuracy in the differentiation between CAO with bronchial involvement (bronchial = 18 and extensive = 20) and tracheal obstruction (n = 12) (Figure 1).

**Table 2:** Pulmonary function tests and lung sound recordings of patients with central airway obstruction.

	Central airway obstruction n = 50		
	Tracheal	Bronchial	Extensive
	n = 12	n = 18	n = 20
Pulmonary function tests			
FVC (l)	2.98±0.34	2.67±0.16	2.32±0.15
(FVC/predFVC, %)	(83.9±6.5)	(80.6±4.0)	(68.0±3.4)*
FEV_1_ (l)	1.09±0.18	1.65±0.11*	1.39±0.12
(FEV_1_/predicted FEV_1_, %)	(39.7±6.9)	(59.6±3.8)**	(49.1±3.4)
FEV_1_/FVC(%)	39.6±6.4	62.7±3.1**	60.1±3.2**
PEF (l/sec)	1.85±0.22	4.54±0.46***	2.75±0.27
Lung sound recordings			
Gap Index (sec)	0.039±0.011	0.216±0.050*	0.190±0.044*

Comparisons between tracheal obstruction group and CAO with bronchial involvement (bronchial and extensive obstruction group) were performed by one-way ANOVA with Dunnett adjustment.

Values are represented as mean ± standard error *p<0.05, **p<0.01, ***p<0.001.

**Figure 2:**
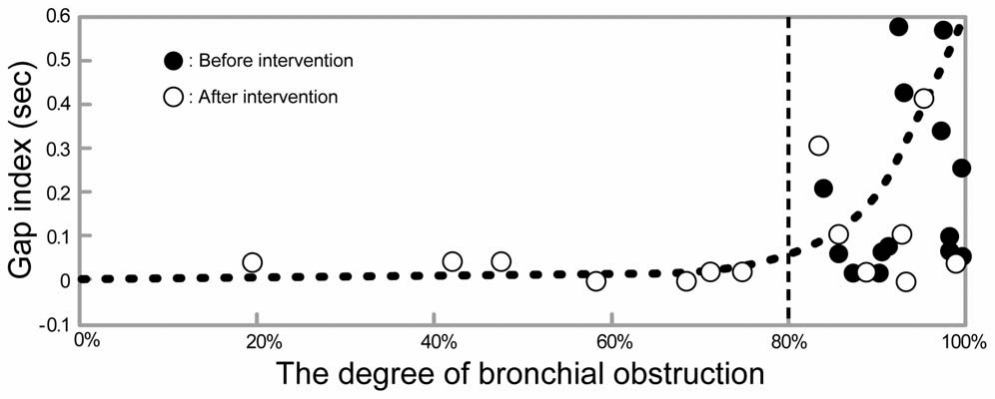
Scatter plot of the gap index versus the degree of bronchial obstruction. *Closed circles, before-intervention; open circles, after-intervention.* This figure shows the correlations between the gap index and the degree of bronchial obstruction in the bronchial obstruction group. The gap index more than 0.06 sec was observed only when the degree of bronchial obstruction was more than 80%. The gap index of cases with 80% or more unilateral bronchial obstruction (0.18±0.18 seconds) was significantly higher than cases with less than 80% obstruction (0.02±0.02 seconds, p<0.05).

After intervention, there were significant improvements in dyspnea for all groups (Table 3; tracheal p<0.01, bronchial p<0.001, extensive p<0.001). In the bronchial obstruction group, no significant improvement was observed in PFTs after intervention. However, the gap index was significantly reduced after intervention (Table 3; −46.2%, p<0.05). In 14 patients with bronchial obstruction, MDCT scans before and after intervention were available and the degrees of bronchial obstruction were analyzed (Table 4). Figure 2 shows a correlation between the gap index and the degree of bronchial obstruction in the bronchial obstruction group. A gap index of more than 0.06 sec was observed only when the degree of bronchial obstruction was more than 80%. The gap index in cases with 80% or more unilateral bronchial obstruction (0.18±0.04 seconds) was significantly higher than that of patients with less than 80% obstruction (0.02±0.01 seconds, p<0.01). The relationship between the baseline of the gap index and the improvement in MMRC in 38 patients with bronchial involvement (bronchial = 18 and extensive = 20) is shown in Table 5. The clinical responder rates were 79.3% for gap indexes over 0.06 seconds and 55.6% for gap indexes of 0.06 seconds or under.

**Table 3:** Dyspnea scale, pulmonary function tests and lung sound recordings before and after intervention.

	Tracheal (n = 12)		Bronchial (n = 18)		Extensive (n = 20)	
	Before	After	Before	After	Before	After
MMRC	3.3±0.3	1.3±0.3**	1.6±0.2	0.8±0.2***	2.6±0.2	1.3±0.2***
Responders		11/12 (92%)		12/18 (67%)		16/20 (80%)
Pulmonary function tests						
FVC (l)	2.98±0.35	3.34±0.30	2.67±0.17	2.90±0.14	2.32±0.15	2.76±0.15**
FEV_1_ (l)	1.09±0.18	2.30±0.25**	1.65±0.11	1.86±0.11	1.39±0.12	1.87±0.12***
PEF (l/sec)	1.85±0.22	4.79±0.47**	4.54±0.46	4.40±0.42	2.75±0.27	3.93±0.37***
Lung sound recordings						
Gap Index (sec)	0.039±0.011	0.070±0.021	0.216±0.050	0.088±0.026*	0.190±0.044	0.124±0.027

Definition of abbreviation: MMRC  =  modified Medical Research Council Scale.

Continuous valuables before and after intervention were tested by Wilcoxon signed-rank test.

Values are represented as mean ± standard error. *p<0.05, **p<0.01, ***p<0.001.

MMRC responder  =  improvement in MMRC scale grade by 1 or more.

**Table 4:** The degree of obstruction and Gap index before and after intervention in cases with bronchial obstruction.

Cases	Degree of obstruction		Gap Index (sec)	
	Before	After	Before	After
63 yr (Male)	99.7%	68.4%	0.057	0
37 yr (Male)	92.3%	88.0%	0.574	0.021
65 yr (Female)	85.6%	71.2%	0.064	0.021
64 yr (Male)	98.1%	58.3%	0.097	0
63 yr (Male)	93.0%	83.5%	0.425	0.306
17 yr (Female)	97.5%	93.1%	0.567	0
62 yr (Male)	99.6%	47.7%	0.255	0.043
57 yr (Male)	87.2%	85.5%	0.021	0.106
62 yr (Male)	90.6%	74.8%	0.064	0.019
45 yr (Male)	90.9%	42.1%	0.073	0.043
26 yr (Male)	89.9%	92.8%	0.019	0.106
71 yr (Female)	83.9%	19.4%	0.208	0.043
25 yr (Female)	99.3%	99.2%	0.038	0.043
61 yr (Female)	96.9%	95.2%	0.34	0.413

**Table 5:** Relation between the baseline of the degree of gap index and the change in MMRC scale after interventional bronchoscopy in cases with bronchial involvement.

Gap index of pre-intervention (sec)	Δ MMRC		
	0	≥1	Responders (%)
0–0.06	4	5	5/9 (55.6%)
0.06<	6	23	23/29 (79.3%)

Definition of abbreviations: MMRC  =  modified Medical Research Council.

Δ MMRC  =  change in MMRC scale.

Responders  =  improvement in MMRC scale by 1 or more.

**Figure 3:**
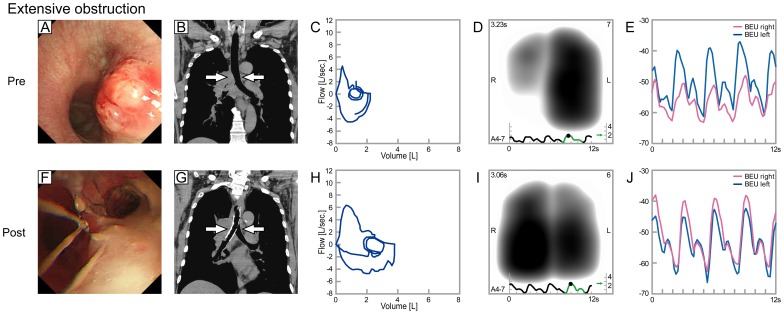
Bronchoscopic and CT findings, flow-volume loops and lung sound recordings for extensive obstruction (before, A–E; after, F–J). This figure shows data of a patient with lung adenocarcinoma. The tumor protrudes from the right side of the trachea and obstructs the right main stem bronchus. The development of lung sound intensity in the right lung, especially the lower lung field, was weaker and lagged behind the left lung (right, red; left, blue). After intervention, these asynchronies were mostly resolved.

Figure 3 represents a patient with lung adenocarcinoma. The tumor protrudes from the right side of the trachea and obstructs the right main stem bronchus. The development of lung sound intensity in the right lung, especially in the lower lung field, was weaker and lagged behind that of the left lung (Lung sound distribution, Video S1 left panel: VRI before intervention; Video S1 right panel: VRI after intervention, online data supplement. The biphasic curve at the bottom stands for inspiratory and expiratory lung sound intensity). After intervention, these asynchronies were mostly resolved.

**Figure 4:**
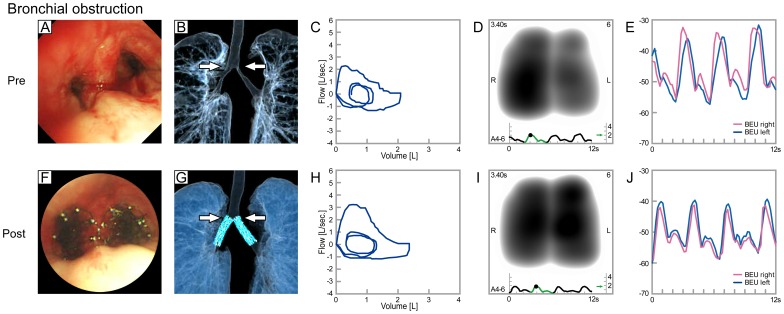
Bronchoscopic and CT findings, flow-volume loops and lung sound recordings for bronchial obstruction (before, A–E; after, F–J). The left bronchus was severely stenotic as a result of lung adenocarcinoma. The development of lung sound intensity in the left lung lagged behind the right lung (right, red; left, blue). After intervention, these asynchronies were mostly resolved.

Figure 4 shows the bronchoscopic, CT and lung sound recordings (Lung sound distribution, Video S2 left panel: VRI before intervention; Video S2 right panel: VRI after intervention, online data supplement) of a patient with lung adenocarcinoma. Her left main stem bronchus was severely obstructed, and the development of lung sound intensity in the left lung lagged behind the right. After intervention, these asynchronies were mostly resolved.

## Discussion

In this study, the left and right lung asynchrony found to be significant at 80% or more bronchial obstruction. These findings revealed that patients with bronchial obstruction and with obvious asynchrony of both lungs displayed a higher responder rate for dyspnea. Furthermore, after intervention for bronchial obstruction, the dyspnea scale and the gap index significantly improved, although the spirometric assessment was unable to show these significant improvements. To our knowledge, this is the first prospective investigation demonstrating the usefulness of left and right lung asynchrony in the diagnosis and treatment of CAO patients.

Although PFTs such as flow-volume curves are important in the assessment of total lung function, PFTs require patient effort and cannot always be performed in CAO patients with severe dyspnea. IOS does not require forced breath maneuvers and provides useful information on collapsible segments. IOS measurements have been proven to correlate with symptomatic improvements after interventional bronchoscopy in tracheal obstruction [14]. However, IOS cannot also discriminate between the left and right bronchial obstructions.

Although we have applied bronchoscopy and three-dimensional computed tomography scans for evaluating bronchial obstructions, non-invasive, reliable and physiological assessment tools for bronchial obstruction are required for outcome assessment after interventional bronchoscopy.

In patients with bronchial obstruction, airflow of the obstructed side lags behind that of the preserved side. This airflow asynchrony in both lungs may be applicable as a physiological indicator in bronchial obstruction. Lung sounds have been used as a surrogate for regional lung airflow [16]–[7]. Recently, Shi et al. reported that measurements of regional ventilation distribution by VRI were comparable to those obtained by electrical impedance tomography [18]. Lung sound recordings are effort-independent, noninvasive, and can be applied even in critical cases [19], [23]. The left and right lung sound asynchrony induced by bronchial obstruction can be evaluated by the gap index. Wang and colleagues reported significant asynchrony between the left and right lungs in asthma exacerbations [25]. They also demonstrated that the asynchrony was significantly reduced, and clinical improvements were noted following treatment.

In this study we found significant asynchrony between the left and right lungs in CAO with bronchial involvement, and patients with a baseline gap index above 0.06 seconds showed a higher responder rate for dyspnea after the intervention. The clear asynchrony in both lungs is thought to be an important finding in planning treatment procedures in CAO patients.

In the outcome assessment of interventional bronchoscopy for bronchial stenosis cases, PFTs may not change significantly after intervention [19]. In this study, the dyspnea scale and the gap index were significantly improved after intervention although there were no significant improvements for PFTs. The evaluation of left and right lung asynchrony may provide a distinct prospective point-of-view in the assessment of interventional outcomes for cases with bronchial obstruction.

Recently, there have been extensive developments in functional imaging related to regional lung function, such as the Xenon ventilation CT [29] and hyperpolarized MRI [30]. These methods are unique and promising due to the high spatial and temporal resolution of respiratory disease morphology and ventilation volumetry. Although the applications of these methods require irradiation or a long acquisition time that limits the accessibility for patients in critical condition at this time, innovation of the functional imaging field will provide ideal methods for evaluating left and right lung asynchrony.

A limitation of this study was the difficulty in sensor attachment to the bony posterior chest wall, which resulted in excluding thinner patients with a BMI of 19 or less. Lower body weight is not uncommon in the Japanese population, especially in the older population. Further improvements in sensor attachment are expected.

## Supporting Information

Video S1 **Lung sound distribution (1).** Left panel: VRI before intervention. Right panel: VRI after intervention. The development of lung sound intensity in the right lung, especially in the lower lung field, was weaker and lagged behind that of the left lung. After intervention, these asynchronies were mostly resolved. The biphasic curve at the bottom stands for inspiratory and expiratory lung sound intensity.
(MP4)

Video S2 **Lung sound distribution (2).** Left panel: VRI before intervention. Right panel: VRI after intervention. The development of lung sound intensity in the left lung lagged behind the right. After intervention, these asynchronies were mostly resolved. The biphasic curve at the bottom stands for inspiratory and expiratory lung sound intensity.
(MP4)
